# Predictive value of iron parameters in neurocritically ill patients

**DOI:** 10.1002/brb3.1163

**Published:** 2018-11-19

**Authors:** Ling Xie, Yu Peng, Kaibin Huang, Yongming Wu, Shengnan Wang

**Affiliations:** ^1^ Department of Neurology, Nanfang Hospital Southern Medical University Guangzhou China

**Keywords:** ferritins, iron, neurocritical care, prognosis

## Abstract

**Background:**

Iron, an essential mineral for human body, has the potential to cause toxicity at high levels. Previous studies have shown inconsistent predictive value of iron parameters in critically ill patients. Thus, we aimed to evaluate the performance of iron parameters in outcome prediction of neurocritically ill patients.

**Methods:**

Retrospective data were collected from patients admitted to the neurocritical care unit (NCU) of a tertiary teaching hospital between August 2016 and January 2017. The iron parameters were obtained at NCU admission. Primary endpoints were short‐term (30‐day) mortality and long‐term (6‐month) poor outcome, with the latter defined as modified Rankin Scale of 4–6. The predictive value of variables was determined with univariate and multivariate logistic analysis. A further subanalysis was conducted in patients stratified by the level of estimated glomerular filtration rate (eGFR).

**Results:**

Of 103 eligible patients, the etiology included stroke (58.2%, *N* = 60), central nervous system infection (13.6%, *N* = 14), and other neurologic disorders (28.2%, *N* = 29). The correlation analysis showed that the increase in ferritin, as well as the reduction in transferrin and total iron‐binding capacity, had strong correlation with C‐reactive protein, procalcitonin, duration of NCU stay, Acute Physiology and Chronic Health Evaluation II score, and Sequential Organ Failure Assessment score. In a further subanalysis of 75 patients with eGFR ≥ 60 ml/min/1.73 m^2^, twelve (16.0%) patients died within 30 days and 39 (52.0%) patients achieved good follow‐up outcome data. In the multivariate logistic regression analysis, we identified baseline ferritin level as an independent predictor of short‐term mortality (OR: 1.002; 95% CI: 1.000–1.003; *p* = 0.008) and long‐term functional outcome (OR: 1.002; 95% CI: 1.000–1.004; *p* = 0.031).

**Conclusions:**

Serum ferritin level at admission could be used as an independent predictor of short‐term mortality and long‐term functional outcome in neurocritically ill patients with eGFR ≥ 60 ml/min/1.73 m^2^.

## 1 INTRODUCTION

Iron has many different roles in the body, acting in carrying oxygen, delivering electron, and catalyzing many biochemistry reactions. After being absorbed from the microvilli of enterocytes, most of the iron in the body keeps a sequestered condition by binding with transferrin in the circulation and is stored in the form of ferritin in the tissues (Andrews & Schmidt, 2007). In contrast, the non‐transferrin‐bound iron is toxic because it can generate free radicals via participating in the Fenton/Haber–Weiss reaction. These reactive oxidative species could cause lipid peroxidation and bring damages to proteins and DNA (Brissot, Ropert, Le Lan, & Loreal, 2012; Koskenkorva‐Frank, Weiss, Koppenol, & Burckhardt, 2013).

Under normal conditions, there is a balance of iron metabolism that preserves the biological function of iron while preventing an excess that would cause oxidative stress. Under pathological conditions, however, the iron metabolism shifts and the alteration could cause it to become a defense mechanism. The processes of iron metabolism involve a number of specific proteins (Malyszko, Malyszko, Pawlak, & Mysliwiec, 2006). Hepcidin, a peptide mainly synthesized by the liver, is regarded as the master regulator modulated in response to hypoxia, iron deficiency, anemia, or inflammation. Iron metabolism disturbance occurs frequently in intensive care unit (ICU) patients, whereby some of the iron parameters reported to be useful in predicting the prognosis of these patients. Nevertheless, these studies are mainly performed in surgical or general ICU. Moreover, which iron parameters could be used to predict reliably with prognosis of critical illness is controversial (Bobbio‐Pallavicini et al., 1989; Leaf, Rajapurkar, Lele, Mukhopadhyay, & Waikar, 2014; Tacke et al., 2016).

In terms of neurologic disorders, several studies (Davalos et al., 1994; Millan et al., 2007; Millerot et al., 2005; Simon et al., 2015; Zuliani et al., 2006) have suggested that baseline ferritin was related to poor outcome and may represent a marker of disease severity, especially in stroke and brain trauma. However, whether iron parameters, especially ferritin, could be used to predict the prognosis in neurocritically ill patients has not been proved yet.

Here, we performed this study to evaluate the predictive value of iron parameters in neurocritically ill subjects.

## 2 METHODS

### 2.1 Subjects

We retrospectively collected data from a prospectively designed cohort of consecutive patients admitted to a neurocritical care unit (NCU) of a tertiary university‐affiliated academic hospital (Nanfang Hospital, Southern Medical University, Guangzhou, China), from August 2016 to January 2017. The inclusion criteria were Glasgow Coma Scale (GCS) (Teasdale & Jennett, 1974) ≤12 and/or admission Acute Physiology and Chronic Health Evaluation (APACHE) II score (Knaus, Draper, Wagner, & Zimmerman, 1985) >15; and/or demand for intensive care or life‐support measures. Patients younger than 18 years old, with iron‐related disorders in history, pregnant, or required intensive care for <72 hr were excluded. The critical stroke patients with severe neurologic deficits, without altered consciousness, and being classified as “demand for intensive care or life‐support measures,” were included in the study. We excluded patients “who required intensive care for <72 hr” because our NCU and our stroke unit worked as the same unit. The patient group with acute mild to moderate intracranial hemorrhage within 24 hr and superacute ischemic stroke with thrombolysis and/or intravascular treatment that required transient close monitoring but transferred out of NCU within 72 hr was eliminated from the neurocritically ill patient group.

Several literatures have reported that iron metabolism may be affected by the renal function and renal function is connected to mortality in ICU patients (Beier et al., 2011; Cartin‐Ceba, Afessa, & Gajic, 2007; Malyszko et al., 2006), indicating that the renal function might have influence on outcome in this study. Therefore, the enrolled patients were divided into two subgroups for further analysis, based on estimated glomerular filtration rate (eGFR) (Levey et al., 2009), with eGFR ≥ 60 ml/min/1.73 m^2^ defined as normal (National Kidney Foundation, 2002).

### 2.2 Data collection

Electronic medical records were carefully reviewed to collect the patient information of demographics, diagnoses, vital signs, GCS scores, APACHE II scores, Sequential Organ Failure Assessment (SOFA) scores (Vincent et al., 1998), laboratory values, length of NCU stay, and duration of mechanical ventilation. Laboratory data of iron parameters included serum iron, ferritin, transferrin, transferrin saturation (TS), total iron‐binding capacity (TIBC), and unsaturated iron‐binding capacity (UIBC). All data mentioned above were referred to the patients’ baseline information within 24 hr after admission. Nobody had prior transfusion. GCS scores were extracted from the first neurological examination at NCU admission. The total scores of APACHE II and SOFA were obtained according to the corresponding parameters within the first 24 hr of NCU admission.

Primary endpoints were short‐term (30‐day) all‐cause mortality and long‐term (6‐month) poor outcome, with the latter defined as modified Rankin Scale (mRS) of 4–6. The outcome information was acquired in a medical follow‐up system, in which all patients admitted to NCU were followed up through face‐to‐face or telephone interviews by a trained personnel blinded to the present study.

### 2.3 Statistical analysis

Continuous data were presented as mean ± standard deviation (*SD*) or median (25%‐75% interquartile range) and compared by Student's *t* test or Mann–Whitney *U* test, as appropriate. Differences in proportions among categorical data were assessed using chi‐squared tests and Fisher's exact tests for multiple groups. The prognostic value of iron parameters was first assessed by univariate analysis. Then, the significant variables were further included in multivariate models for adjustment. The 95% confident intervals reported for the logistic regression odds ratios were calculated by the maximum‐likelihood estimation (forward selection). Correlations between variables were determined with the Spearman's rank correlation test. *p* < 0.05 was considered statistically significant. All statistical analyses were performed using SPSS, version 20.0 (SPSS, Chicago, IL).

### 2.4 Ethic statement

The study proposal was approved by the Medical Ethics Committee of Nanfang Hospital. Informed consents were signed by all the patients or their surrogates before data collection.

## 3 RESULTS

### 3.1 Overall analysis

**Figure 1:**
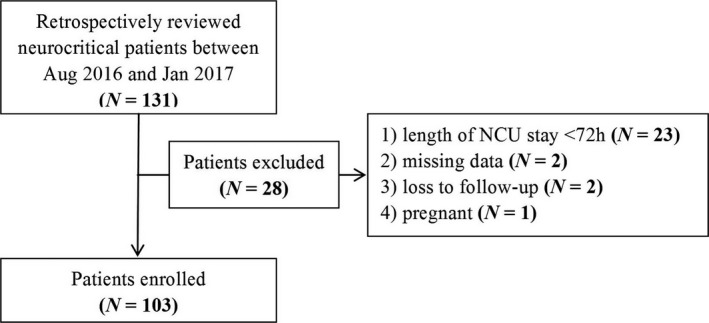
Patient inclusion flowchart. NCU: neurocritical care unit

Of 131 patients screened for eligibility, 103 satisfied inclusion and exclusion criteria (Figure 1). The etiology of the patients included acute ischemic stroke (38.8%, *N* = 40), intracranial hemorrhage (19.4%, *N* = 20), central nervous system infection (13.6%, *N* = 14), and other neurologic disorders (28.2%, *N* = 29).

For short‐term mortality, higher baseline ferritin (*p* = 0.017) and lower baseline transferrin (*p* = 0.018) and TIBC (*p* = 0.037) showed statistical significance. For long‐term prognosis, baseline transferrin was the only iron parameter showed statistical significance (*p* = 0.043) (Table 1). In multivariate logistic regression model, none of the iron parameters showed statistical significance (not shown).

**Table 1:** Characteristics of studied NCU patients

Parameters	Short‐term mortality			Long‐term poor functional outcome		
	No (*N* = 81)	Yes (*N* = 22)	*p*	No (*N* = 45)	Yes (*N* = 58)	*p*
Age (years, median, IQR)	57.0 (43.0,67.0)	65.5 (38.8,72.8)	0.087	54.0 (38.5,66.5)	61.5 (43.5,71.0)	0.057
Male (*n*, %)	49 (60.5)	14 (63.6)	0.789	28 (62.2)	35 (60.3)	0.846
Primary NCU diagnosis (*n*, %)			0.202			0.266
Stroke	48 (59.3)	12 (54.6)		25 (55.6)	35 (60.4)	
CNS infections	13 (16.0)	1 (4.5)		9 (20.0)	5 (8.6)	
Other neurologic disorders	20 (24.7)	9 (40.9)		11 (24.4)	18 (31.0)	
Prior history (*n*, %)						
Hypertension	46 (56.8)	14 (63.6)	0.564	25 (55.6)	35 (60.3)	0.625
Diabetes	19 (23.5)	9 (40.9)	0.103	9 (20.0)	19 (32.8)	0.149
Length of NCU stay (days, median, IQR)	5.0 (3.0,12.0)	7.5 (4.5,16.3)	0.277	4.0 (3.0,6.0)	8.5 (4.8,17.0)	<0.001*
APACHE II score (mean ± *SD*)	11.6 ± 5.6	18.0 ± 6.9	<0.001*	10.0 ± 5.5	15.2 ± 6.1	<0.001*
SOFA score (median, IQR)	4.0 (3.0,6.0)	10.0 (8.8,12.0)	<0.001*	3.0 (2.0,5.5)	8.0 (5.0,10.0)	<0.001*
eGFR (ml/min/1.73 m^2^, mean ± *SD*)	86.3 ± 33.9	67.6 ± 38.8	0.009*	94.0 ± 28.4	73.2 ± 38.1	0.002*
C‐reactive protein (mg/dl, median, IQR)	15.3 (5.2,58.4)	42.3 (8.8,132.0)	0.070	8.9 (3.7,54.5)	32.7 (10.6,94.3)	0.009*
Procalcitonin (μg/L, median, IQR)	0.123 (0.054,0.494)	0.461 (0.118,2.560)	0.006*	0.098 (0.050,0.366)	0.201 (0.075,0.830)	0.024*
Iron parameters						
Ferritin (ng/ml, median, IQR)	322.5 (170.9,634.6)	583.2 (294.7,1066.2)	0.017*	336.8 (169.1,597.4)	393.3 (215.6,866.5)	0.107
Serum iron (μmol/L, median, IQR)	7.0 (4.3,10.0)	5.4 (4.0,8.0)	0.198	8.0 (4.2,12.0)	6.0 (4.2,8.0)	0.070
UIBC (μmol/L, mean ± *SD*)	31.83 ± 9.38	28.55 ± 13.67	0.299	32.28 ± 9.25	30.23 ± 11.29	0.326
TIBC (μmol/L, mean ± *SD*)	39.77 ± 10.30	35.43 ± 12.55	0.037*	41.02 ± 10.81	37.16 ± 10.76	0.074
TS (%, median, IQR)	17.6 (12.2,25.7)	16.7 (11.8,25.3)	0.661	19.0 (13.2,26.8)	16.7 (11.5,24.3)	0.277
Transferrin (g/L, median, IQR)	1.82 (1.51,2.14)	1.39 (1.20,2.15)	0.018*	1.85 (1.63,2.14)	1.62 (1.32,2.15)	0.043*

APACHE: Acute Physiology and Chronic Health Evaluation; CNS: central nervous system; eGFR: estimated glomerular filtration rate; IQR: interquartile range; NCU: neurocritical care unit; *SD*, standard deviation; SOFA: Sequential Organ Failure Assessment; TIBC: total iron‐binding capacity; TS: transferrin saturation; UIBC: unsaturated iron‐binding capacity.

^*^ *p* < 0.05.

The correlation analysis showed that the increase in ferritin, as well as the reduction in transferrin and TIBC, was paralleled with the increase in duration of NCU stay, APACHE II scores, SOFA scores, C‐reactive protein, and procalcitonin levels (Table 2).

**Table 2:** Correlations between iron parameters and selected variables in total neurocritically ill patients

Parameters	Duration of NCU stay		APACHE II score		SOFA score		C‐reactive protein		Procalcitonin	
	*r*	*p*	*r*	*p*	*r*	*p*	*r*	*p*	*r*	*p*
Ferritin	0.229	0.020	0.281	0.004	0.378	<0.001	0.453	<0.001	0.331	0.001
Serum iron	‐	NS	−0.244	0.013	−0.204	0.038	−0.527	<0.001	−0.317	0.001
UIBC	‐	NS	−0.233	0.018	−0.258	0.009	−0.321	0.001	−0.356	<0.001
TIBC	−0.200	0.043	−0.316	0.001	−0.323	<0.001	−0.508	<0.001	−0.437	<0.001
TS	‐	NS	‐	NS	‐	NS	−0.270	0.006	‐	NS
Transferrin	−0.196	0.048	−0.348	<0.001	−0.362	<0.001	−0.516	<0.001	−0.488	<0.001

APACHE: Acute Physiology and Chronic Health Evaluation; NCU: neurocritical care unit; NS: not significant; SOFA: Sequential Organ Failure Assessment; TIBC: total iron‐binding capacity; TS: transferrin saturation; UIBC: unsaturated iron‐binding capacity.

### 3.2 Subgroup analysis

To eliminate the confounding of renal function, we performed subgroup analysis in patients with eGFR ≥ 60 ml/min/1.73 m^2^ (*N* = 75).

### 3.3 30‐day mortality of patients with eGFR ≥ **60 ml/min/1.73 m^2^**

Compared with short‐term survivors (63 patients), the victims were more likely to have underlying diabetes, higher APACHE II and SOFA scores at admission, whereas there were no differences in any iron parameters (Supporting information Table S1). In multivariate logistic analysis, ferritin was independently associated with 30‐day mortality (OR: 1.002; 95% CI: 1.000–1.003; *p = *0.008) (Table 3).

**Table 3:** Univariate and multivariate logistic regression analysis for iron parameters on adverse outcomes in NCU patients with eGFR ≥ 60 ml/min/1.73 m^2^

	Short‐term mortality		Long‐term poor functional outcome	
	OR (95% CI)	*p*	OR (95% CI)	*p*
Univariate analysis				
Ferritin	1.002 (1.000–1.003)	0.022	1.001 (1.000–1.003)	0.044
Serum iron	‐	0.689	‐	0.391
UIBC	‐	0.383	‐	0.629
TIBC	‐	0.291	‐	0.366
TS	‐	0.592	‐	0.937
Transferrin	‐	0.188	‐	0.311
Multivariate analysis				
Ferritin	1.002 (1.000–1.003)	0.008	1.002 (1.000–1.004)	0.031

Note

These factors were adjusted in multivariate regression models: age, gender, etiology, diabetes, and Acute Physiology and Chronic Health Evaluation (APACHE) II score.

eGFR: estimated glomerular filtration rate; NCU: neurocritical care unit; TIBC: total iron‐binding capacity; TS: transferrin saturation; UIBC: unsaturated iron‐binding capacity.

### 3.4 6‐month functional outcome of patients with eGFR ≥ 60 ml/min/1.73 m^2^

Increased APACHE II score, SOFA score, length of NCU stay, and serum C‐reactive protein levels were observed in patients with poor functional outcome at 6 months compared to those with good functional outcome (39 patients), while there were no differences in any iron parameters (Supporting information Table S1). In multivariate logistic analysis, ferritin was independently associated with 6‐month functional outcome (OR: 1.002; 95% CI: 1.000–1.004; *p = *0.031) (Table 3).

## 4 DISCUSSION

In this study, we verified serum ferritin as a predictor of clinical outcome in a cohort of neurocritically ill patients and found that serum ferritin was independently associated with 30‐day mortality and 6‐month poor functional outcome in neurocritically ill patients with eGFR ≥ 60 ml/min/1.73 m^2^.

Our results showed that neurocritically ill patients who died within 30 days tended to have higher baseline serum ferritin as well as lower transferrin and TIBC, and those with poor functional outcome at 6 months presented with a lower transferrin level at admission. These results were in line with former studies. Darveau, Denault, Blais, & Notebaert (2004) found that elevated ferritin and diminished serum iron, TS, and transferrin levels are generally observed in more critically ill patients. Bobbio‐Pallavicini et al. (1989) investigated 51 postoperative critically ill patients and found that the increase in ferritin concentration correlated with worsening of the clinical status. In a prospective observational cohort study of 121 critically ill patients, plasma catalytic iron levels on ICU day 1 were significantly associated with hospital mortality and 30‐day mortality (Leaf et al., 2014). These results could be explained by the common inflammation, especially infectious inflammation, in critically ill patients. In the presence of inflammation, a great amount of hepcidin is released, which subsequently degrades the cellular iron exporter ferroportin on enterocytes and macrophages and limits the availability of serum iron, resulting in decreased TS. Pro‐inflammatory cytokines, such as interleukin (IL)‐1β, IL‐6, and tumor necrosis factor, induce the transcription and translation of ferritin, resulting in increased ferritin. Intracellular iron content modulates the binding affinity of cytoplasmic iron regulatory proteins (IRP)‐1 and IRP‐2 with iron‐responsive elements and rapidly reduces the mRNA expression of transferrin receptors causing transferrin concentrations decreased. Our study also demonstrated the serum iron, transferrin, UIBC, TIBC, and ferritin levels had significant correlation with APACHE II scores and infectious biomarkers at admission.

In subgroup analysis, we demonstrated serum ferritin was an independent predictor of short‐term mortality and long‐term poor outcome, in agreement with previous researches in ICU patients (Leaf et al., 2014; Munoz et al., 2005; Simon et al., 2015; Tacke et al., 2016). And in neurologic disorders, Millan et al. (2007) studied 134 consecutive patients with acute ischemic stroke and found that ferritin levels before thrombolytic treatment were higher in patients with poor outcome (modified Rankin scale, mRS > 2) at 90 days. It was suspected that increased ferritin could be in part the result of a neuroprotective mechanism with the aim of sequestering toxic‐free iron in the ischemic brain. Another study performed in patients with intracerebral hemorrhage also demonstrated that serum ferritin at baseline was markedly higher and iron as well as transferrin was lower in patients with poor outcome (mRS ≥ 3) at 90 days (Yang et al., 2016). A prospective study enrolled 69 male patients who suffered from severe traumatic brain injury, and the results showed that higher ferritin concentrations were significantly associated with fatal outcome (Simon et al., 2015). Ferritin is known as an acute‐phase protein in response to inflammation, a common pathological state in critically ill patients (Marshall, 2001). Here, we also observed that the ferritin levels were moderately correlated with the infectious biomarkers and APACHE II and SOFA scores in this study. Infectious inflammation stimulates the upregulated expression of hepcidin and ferritin, resulting in relative iron deficiency in bloodstream (Drakesmith & Prentice, 2012). Upon that the ferritin level as the hallmark of iron homeostasis fluctuation probably reflects the severity of inflammatory response and risks of multiple organ dysfunction and anemia, complications that contribute to the increased mortality in critical illness. In this study, we found for each 10 ng/ml raise in ferritin, there was 2% increase in odds of short‐term mortality.

Since early evaluation of neurocritically ill patients at risk of mortality may benefit the management of their treatment strategies, the protective effect of intervention in disturbances of iron homeostasis in neurocritical illness might worthy to be further investigated (Ganz, 2013; Ma, You, & Hao, 2012; Selim, 2009).

Several limitations of this study should be noted. First, the retrospective nature of this study makes it susceptible to selection and information bias. Second, we were unable to assess all potentially relevant variables. Third, the sample size of this study was relatively small. Further studies should be done to validate the results, and the impact of treatment strategies for iron metabolism on the prognosis is promising.

In conclusion, our study demonstrated that elevated ferritin level was independently associated with increased short‐term mortality and long‐term poor outcome in neurocritically ill patients with eGFR ≥ 60 ml/min/1.73 m^2^. Future studies with larger sample size should be conducted to confirm our conclusions, and the protective effect of intervention on iron metabolism among neurocritically ill patients might deserve further investigation.

## CONFLICT OF INTEREST

None declared.
