# Supplementary material for: Predictive value of iron parameters in neurocritically ill patients
Source: Brain Behav. 2018 Nov 19;8(12):e01163. doi: 10.1002/brb3.1163 (PMC6305919; doi:10.1002/brb3.1163)
Supplement: Supplementary file 1 [file BRB3-8-e01163-s001.docx]

**TABLE S1** Characteristics of studied NCU patients with eGFR ≥ 60 mL/min/1.73m²

| **Parameters** | **Short-term mortality** | | | **Long-term poor functional outcome** | | |
| --- | --- | --- | --- | --- | --- | --- |
|  | **No (*N* = 63)** | **Yes (*N* = 12)** | ***p*** | **No (*N* = 39)** | **Yes (*N* = 36)** | ***p*** |
| Age (years, median, IQR) | 53.0(40.0,64.0) | 59.5(33.3,66.0) | .583 | 53.0(35.0,64.0) | 57.0(41.3,64.3) | .436 |
| Male (n, %) | 37(58.7) | 8(66.7) | .847 | 23(59.0) | 22(61.1) | .850 |
| Primary NCU diagnosis (n, %) |  |  | .572 |  |  | .213 |
| Stroke | 35(55.5) | 6(50.0) |  | 20(51.3) | 21(58.4) |  |
| CNS infections | 11(17.5) | 1(8.3) |  | 9(23.1) | 3(8.3) |  |
| Other neurologic disorders | 17(27.0) | 5(41.7) |  | 10(25.6) | 12(33.3) |  |
| Prior history (n, %) |  |  |  |  |  |  |
| Hypertension | 34(54.0) | 6(50.0) | .801 | 22(56.4) | 18(50.0) | .578 |
| Diabetes | 14(22.2) | 7(58.3) | .028* | 9(23.1) | 12(33.3) | .323 |
| Length of NCU stay (days, median, IQR) | 5.0(3.0,11.0) | 10.5(5.3,17.0) | .064 | 4.0(3.0,6.0) | 9.5(4.3,16.8) | .002* |
| APACHE II score (mean±SD) | 10.3±5.1 | 14.2±7.0 | .027* | 9.0±5.1 | 12.9±5.5 | .003* |
| SOFA score (median, IQR) | 4.0(2.0,6.0) | 9.5(6.0,10.8) | .001* | 3.0(2.0,5.0) | 6.0(4.0,10.0) | <.001* |
| eGFR (mL/min/1.73m², mean±SD) | 100.7±21.1 | 93.3±34.0 | .102 | 102.0±20.4 | 96.8±26.5 | .185 |
| C-reactive protein (mg/dL, median, IQR) | 11.2(3.8,52.8) | 34.9(8.2,141.3) | .152 | 7.9(2.9,40.2) | 30.1(9.6,82.0) | .013* |
| Procalcitonin (μg/L, median, IQR) | 0.082(0.049,0.319) | 0.139(0.092,0.875) | .059 | 0.082(0.048,0.220) | 0.117(0.064,0.584) | .143 |
| Iron parameters |  |  |  |  |  |  |
| Ferritin (ng/mL, median, IQR) | 322.5(167.0,553.0) | 520.3(205.1,1593.8) | .070 | 257.8(167.0,501.2) | 420.5(186.0,857.7) | .069 |
| Serum iron (μmol/L, median, IQR) | 8.0(4.3,11.0) | 6.0(4.3,8.0) | .314 | 8.0(4.1,13.0) | 6.7(5.0,9.0) | .451 |
| UIBC (μmol/L, mean±SD) | 32.59±8.83 | 29.80±15.91 | .566 | 32.68±8.54 | 31.55±11.83 | .639 |
| TIBC (μmol/L, mean±SD) | 41.11±9.59 | 37.67±14.11 | .433 | 41.60±10.27 | 39.43±10.59 | .372 |
| TS (%, median, IQR) | 18.6(13.2,26.5) | 16.9(11.5,25.0) | .649 | 19.0(12.8,27.1) | 17.6(12.3,25.0) | .668 |
| Transferrin (g/L, median, IQR) | 1.86(1.63,2.16) | 1.45(1.20,2.21) | .314 | 1.86(1.69,2.16) | 1.83(1.37,2.17) | .318 |

IQR, interquartile range; SD, standard deviation; NCU, neurocritical care unit; CNS, central nervous system; APACHE, Acute Physiology and Chronic Health Evaluation; SOFA, Sequential Organ Failure Assessment; eGFR, estimated glomerular filtration rate; UIBC, unsaturated iron-binding capacity; TIBC, total iron-binding capacity; TS, transferrin saturation.

* *p* < .05.
